# Review on Soil Corrosion and Protection of Grounding Grids

**DOI:** 10.3390/ma17020507

**Published:** 2024-01-20

**Authors:** Jing Zhao, Xian Meng, Xiao Ren, Shengfang Li, Fuhao Zhang, Xiaofang Yang, Junyao Xu, Yuan Yuan

**Affiliations:** 1 State Grid Chongqing Electric Power Research Institute, Chongqing 401123, China; zhaojing2@cq.sgcc.com.cn (J.Z.); mengxian@cq.sgcc.com.cn (X.M.); renxiao@cq.sgcc.com.cn (X.R.); 13983688799@139.com (S.L.); 2 College of Materials Science and Engineering, Chongqing University, Chongqing 400044, China; fuzhangh@163.com (F.Z.); yangxf@cqu.edu.cn (X.Y.); yuany@cqu.edu.cn (Y.Y.)

**Keywords:** grounding grid, soil corrosion, corrosion mechanism, corrosion-protection measures

## Abstract

The corrosion of grounding grid materials in soil is a prominent factor in power and electrical equipment failure. This paper aims to delve into the corrosion characteristics of grounding grid materials and the corresponding methods of safeguarding against this phenomenon. Firstly, the influencing factors of the soil environment on the corrosion of the grounding grid are introduced, including soil physicochemical properties, microorganisms, and stray currents. Then, the corrosion behavior and durability of common grounding grid materials such as copper, carbon steel, and galvanized steel are discussed in detail and compared comprehensively. In addition, commonly used protective measures in China and outside China, including anti-corrosion coatings, electrochemical protection, and other technologies are introduced. Finally, it summarizes the current research progress and potential future directions of this field of study.

## 1. Introduction

A grounding grid is an important device used in substations for work grounding, lightning grounding, and protective grounding, to ensure the safety of people, equipment, and systems [1,2,3]. In the case of a lightning strike or power system failure that releases a large amount of current to the ground, the grounding grid plays a role in quickly dissipating the current and reducing the contact voltage and step voltage [4,5]. It can effectively safeguard the stable operation of the power system and enhance the safety of personnel and equipment.

**Figure 1:**
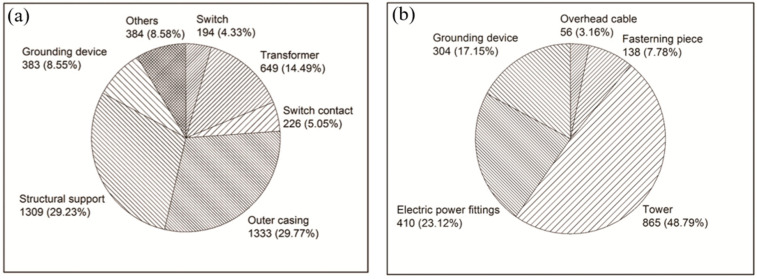
(**a**) Number of corrosion cases of power transformation equipment. (**b**) Number of corrosion cases of power transmission equipment [11].

With the development of the power industry, the safety requirements for substations have become increasingly stringent, demanding higher levels of thermal stability and corrosion resistance for the grounding systems. As a crucial component within the power system, grounding materials are buried in the complex environment of the soil medium for a long period. They are subjected to various corrosive influences, including battery corrosion, soil-composition corrosion, and microbial corrosion [6,7,8]. Steel is employed for the majority of the grounding materials in the system. Carbon steel material becomes brittle, laminating, loose, and even fractured in several places after corrosion begins. The surface is riddled with corrosion pits, which frequently exhibit localized corrosion patterns [9]. Electrochemical corrosion within the soil environment and the corrosion caused by the drain current in the operation of power grid equipment cause the reduction of the cross section of the grounding body and even fractures. As a consequence, the grounding performance is compromised, resulting in an elevated grounding-resistance value, and a diminished capacity to dissipate current. This leads to a local potential difference in the grounding system itself that exceeds the safety value [10]. In addition to posing risks to operators’ safety, secondary equipment’s insulation may be damaged due to backlash or cable skin circulation. The ingress of high voltage into the control room has the potential to devastate monitoring equipment, resulting in significant economic losses and profound social consequences. Xia et al. [11] conducted an investigation of 135 substations and 135 transmission lines with a voltage rating of 110 kV and above, as presented in Figure 1. The occurrence of corrosion in grounding devices was found to be 8.6% and 17.1% in these substations and transmission lines, respectively. Hence, it becomes imperative to emphasize the significance of corrosion protection for the grounding grid and strive for continuous enhancement of the corrosion-protection mechanism and measures. By doing so, the operational efficiency and safety of the power system can be augmented.

This paper provides a comprehensive review of the soil corrosion in a substation grounding grid, along with the associated corrosion issues they encounter. It delves into the factors that influence soil corrosion and discusses common anti-corrosion measures. Furthermore, the paper describes diagnostic techniques and monitoring methods employed to assess the corrosion state of a grounding grid. Lastly, the paper offers suggestions and future perspectives on corrosion protection for a grounding grid.

## 2. Analysis of Research Hotspots and Frontiers in the Field of Soil Corrosion

The analysis of research hotspots and frontiers in the field of soil corrosion is crucial for understanding the progress and development trends in this area, both at domestic and international levels. To provide an objective reflection of these trends, this paper utilizes bibliometric methods to conduct an analysis. By examining the annual trends of soil corrosion papers, the main publishing countries, institutions, authors, and research hotspots and frontiers, this analysis aims to offer valuable references for researchers working in this field.

**Figure 2:**
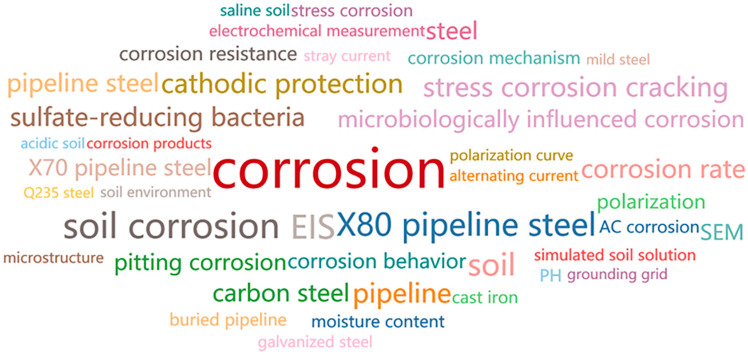
High-frequency keywords for WOS papers in the field of soil corrosion.

Following a thorough search of 821 articles pertaining to soil corrosion in WOS, we proceeded to extract the keywords provided by the authors and conducted a statistical analysis of their frequency. This procedure led us to identify the keywords that appeared 20 times or more, as illustrated in Figure 2. The figure clearly depicts that the primary areas of focus within soil-corrosion research encompass corrosion studies, electrochemical impedance spectroscopy, cathodic protection, and pipeline-steel corrosion.

**Figure 3:**
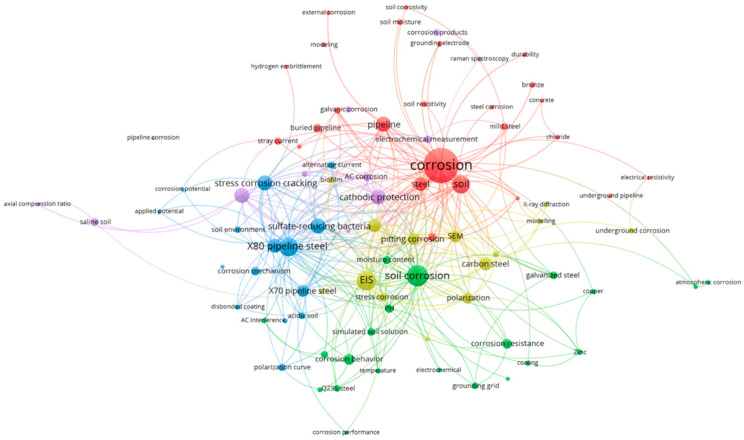
Distribution of hotspots in soil-corrosion-research field based on keyword co-occurrence in WOS papers.

To unveil the distribution of hotspots within the research field of soil corrosion, a network clustering analysis was conducted using the extracted keywords from WOS. The outcomes of this analysis are presented in Figure 3. The size of each circle corresponds to the number of published papers, while the connecting lines between different organizations signify related studies. Notably, Figure 2 illustrates that the primary research areas in soil corrosion have predominantly revolved around factors such as water content and resistivity, with comparatively less attention given to corrosion mechanisms and the operating conditions of underground metals.

## 3. Grounding Grid Materials

In situations where there is a short-circuit in the electrical current, a significant amount of energy is converted into heat within the grounding conductor. However, challenges within the power system often arise rapidly, hindering the dissipation of this generated heat into the surrounding environment. Therefore, it becomes crucial to utilize a substance with exceptional thermal and electrical conductivity properties to absorb this thermal energy within the grounding material. Furthermore, the selected material for the grounding grid must possess substantial mechanical strength and excellent corrosion resistance. These characteristics ensure the grid’s ability to withstand external forces and environmental impacts, maintaining its stable shape and structure while effectively countering the corrosive effects of soil, water, and other external factors.

**Figure 4:**
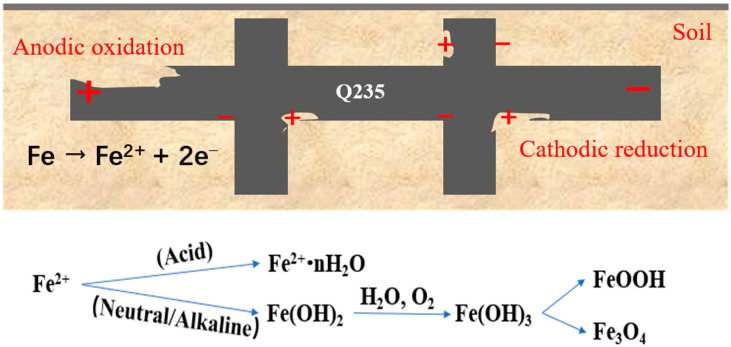
Carbon steel corrosion mechanism in soil.

*Carbon steel.* A multitude of substations in China commonly employ carbon steel as the chosen material for their grounding grids due to its noteworthy yield and tensile strength. Carbon steel demonstrates an ability to withstand significant stresses and pressures, while also offering simplicity in manufacturing and welding processes, as well as adaptability to various shapes and sizes [12,13]. The primary driving factor behind this choice is the comparative affordability and accessibility of steel in contrast to copper. However, it is crucial to note that carbon steel is not optimal for use as a grounding grid material. Its corrosion resistance falls short when compared to copper and galvanized steel [14]. The inherent high electrical resistivity of carbon steel leads to an increased grounding resistance within the grid, thereby hindering the achievement of an optimal discharge effect. Another drawback of carbon steel lies in its relatively lower thermal stability compared to copper. The carbon present in carbon steel can initiate the formation of corrosion microcells, leading to erosion of the grounding body and internal structure. Soil parameters such as soluble salts, pH value, water content, oxygen concentration, and resistivity strongly influence the corrosion process of carbon steel in the soil [15,16,17,18]. The joint parts of the carbon steel grounding grid are generally connected using high-temperature arc welding. However, due to the structural disparity between the welded junction and the underlying material, corrosion susceptibility arises. Carbon steel corrosion in soil primarily manifests as localized corrosion [19], as illustrated in Figure 4.

**Figure 5:**
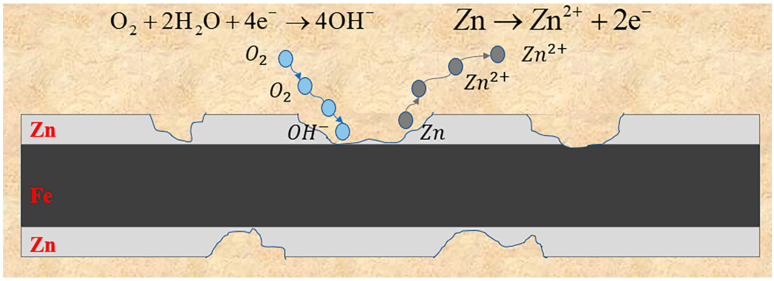
Corrosion mechanism of galvanized steel.

*Galvanized steel.* Galvanized steel refers to carbon steel that has been coated with a layer of zinc. This process involves either electroplating or hot-dip plating the zinc onto the surface of the carbon steel. It presents several advantages such as its simplicity, low cost, and strong conductivity. Consequently, it has become the most prevalent method in China for safeguarding grounding grids. The galvanized layer, which forms a dense oxide film, serves as protection for the underlying steel matrix [20,21]. As zinc possesses a lower electrode potential than carbon steel, a primary cell is formed between the zinc layer and the carbon steel. In this electrochemical setup, zinc is consumed through oxidation as the negative electrode, while the positive electrode, carbon steel, benefits from protective reduction [22]. Empirical evidence supports the notion that the galvanized layer plays a crucial role in safeguarding the underlying carbon steel base [23,24]. However, when galvanized steel is employed as a grounding material, its corrosion resistance only offers marginal enhancement compared to ordinary carbon steel. Over time, the zinc layer begins to corrode, rendering the corrosion products incapable of preserving the underlying structure. This susceptibility arises due to the limited thickness of the galvanized layer and potential defects during the hot-dip-plating process. Subsequent to the damage of the zinc layer, deleterious anions come into contact with the substrate, drastically accelerating the corrosion of the substrate [7,25,26]. As the exposed area of the substrate expands, the galvanized layer becomes incapable of protecting it, leading to equivalent corrosion rates between galvanized steel and carbon steel when placed in soil. Consequently, as corrosion progresses, the substrate suffers rapid deterioration. The corrosion mechanism of galvanized steel is shown in Figure 5.

*Copper.* Copper serves as a widely used grounding material in Europe and America, owing to its extensive usage. It has exceptional conductivity and heat resistance, along with favorable discharge characteristics and thermal stability [27,28,29]. The average annual corrosion rate of copper typically falls below 0.03 mm per year, while the maximum annual pitting rate remains beneath 0.2 mm per year. With its elevated corrosion potential, copper seldom assumes the role of a dissolved anode in electrochemical corrosion scenarios involving other metals or alloys. Instead, it tends to corrode the neighboring metal or alloy while preserving its own structural integrity. Moreover, when exposed to oxygen, the surface of copper undergoes chemical reactions that yield corrosion products, such as basic copper carbonate (Cu_2_(OH)_2_CO_3_). Copper basic carbonate can form a tightly adhered layer on copper surfaces, forming a dense layer of corrosion products. This layer effectively impedes the diffusion of aggressive ions from the soil towards the substrate, as well as the diffusion of corrosion products back into the soil [30,31]. Consequently, the protective capability of the substrate is enhanced, ensuring the prevention of additional corrosion to the internal copper [32]. Copper corrosion exhibits robust durability and is devoid of pitting tendencies, endowing it with a long lifespan. However, when copper is employed as a grounding material, it is prone to interacting with subterranean steel components, leading to the formation of a corrosive primary battery. Copper also corrodes in strongly acidic environments [33]. Wu et al. [34] studied the corrosion behavior of copper in acidic soil. It was found that the corrosion rate of copper accelerated with the increase in soil acidity. In neutral or alkaline environments, copper surpasses galvanized steel in terms of corrosion resistance, showcasing noteworthy advantages. Nonetheless, as a non-ferrous metal, copper confronts challenges related to limited resources, elevated costs, and substantial engineering expenses. The corrosion of copper engenders the release of heavy-metal ions, which pose environmental hazards by contaminating soil and groundwater. These ions can also accumulate within the food chain, indirectly or directly posing risks to human health.

*Copper-plated steel.* The high price of copper in the market coupled with resource shortage has spurred the development of copper-plated steel materials, which exhibit properties comparable to pure copper but come at significantly reduced costs [35]. The copper-plated-steel material entails the application of a specialized process to overlay a copper layer of a certain thickness onto the surface of carbon steel. Notably, the corrosion resistance of copper-coated steel surpasses that of both galvanized steel and carbon steel grounding materials [36]. When exposed to oxygen, similar to copper, copper-plated steel surfaces generate a protective copper oxide–alkali carbonate coating, effectively impeding further corrosion of the underlying carbon steel. Additionally, the electrical conductivity of copper-plated steel is superior to that of carbon steel, and approximately twice that of galvanized steel. Consequently, it allows for a reduction in the cross-sectional area of grounding rods while effectively minimizing grounding resistance. Furthermore, copper-plated steel exhibits commendable mechanical strength. Within an equivalent cross-section, the tensile strength of copper-plated steel rods can exceed 600 MPa, which is twice as high as that of solid copper rods. Consequently, copper-plated steel provides exceptional load-bearing capacity and impact resistance, simplifying construction efforts [37,38]. However, it is crucial to note that due to the limited thickness of the copper-plated-steel plating, any damage to the plating can initiate a galvanic corrosion battery, accelerating the corrosion of the internal steel core. As a result, the utilization of this material remains limited in China, mainly due to insufficient operational experience. Table 1 presents a comprehensive comparison of the overall performance of various grounding materials.

## 4. Factors Affecting Soil Corrosion of Grounding Materials

**Figure 6:**
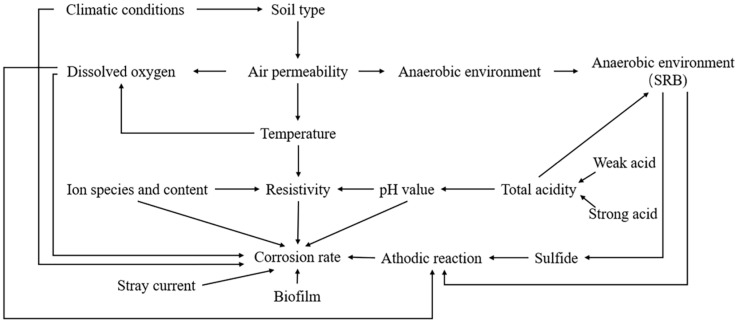
Relationship between factors and soil erodibility.

Soil is a complex system consisting of substances across gaseous, liquid, and solid states. Various factors exert an influence on the corrosive nature of soil, comprising its physical and chemical attributes, electrochemical properties, microorganisms, stray currents, as well as climatic considerations such as temperature [8,39,40,41]. The interconnectedness of these factors mandates a comprehensive evaluation approach in assessing the corrosiveness of soil. Figure 6 illustrates the intricate interplay among the different components contributing to soil corrosion.

### 4.1. Moisture Content

**Figure 7:**
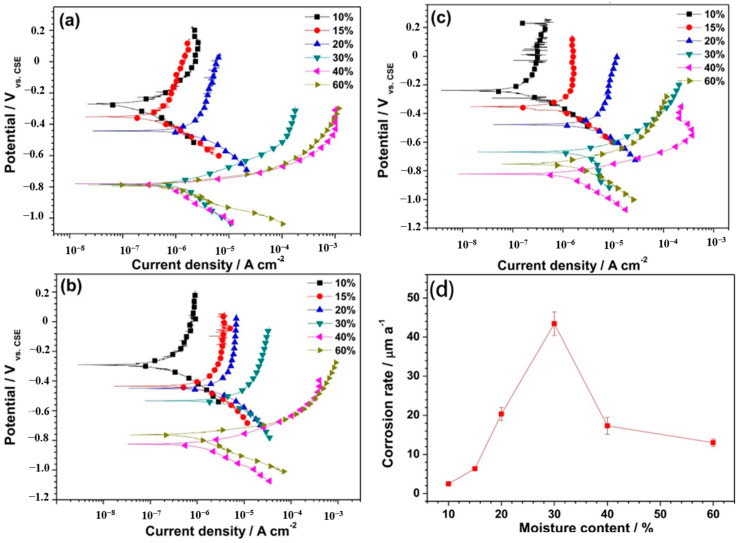
Polarization curves of Q235 steel in the Na-based bentonite clays at different moisture contents over time: (**a**) 240 h; (**b**) 720 h; (**c**) 1080 h. (**d**) Average corrosion rate of Q235 steel soaked in the Na-based bentonite clay for 1080 h with different moisture contents [46].

Grounding grid corrosion primarily manifests as electrochemical corrosion, with soil moisture content emerging as a crucial determinant of such corrosion behavior. Moisture in the soil acts as an electrolyte, providing an environment conducive to the formation of corrosion cells. In addition, alterations in moisture content profoundly impact the physical and chemical properties of the soil, thereby influencing the corrosion tendencies observed in grounding grids [21,42,43]. Noor and Al-Moubaraki [44] investigated the corrosion rate of X60 steel under different water-content conditions. It was found that the corrosion rate increases with increasing water content, and the corrosion rate decreases with further increases in water content after the water content reaches 10 wt%. In a weightlessness experiment conducted by Jing Fu et al. [45], it was found that the peak corrosion of 20G and Q235 galvanized steel occurred at the soil humidity levels of 10% and 12.5%, respectively. Liu et al. [46] investigated the corrosion behavior of Q235 steel in soils with different water contents. As shown in Figure 7, it was found that the corrosion potential of steel decreases and the corrosion rate and current density increase with increasing soil water content. At moisture contents above 30 wt%, the corrosion rate decreases as the moisture content increases. El-Shamy et al. [47] delved into an investigation of mild steel corrosion in clay with different moisture contents and discovered that the corrosion rate increases as the moisture content rises, provided that the content remains below 40%. Once the water content surpasses a critical value, soluble salts saturate the soil. At this critical juncture, optimal levels of oxygen diffusion and soil moisture culminate in a peak corrosion rate. Subsequently, the availability of oxygen to the metal surface diminishes, resulting in a decline in the corrosion rate [48].

### 4.2. Soil pH Value

**Figure 8:**
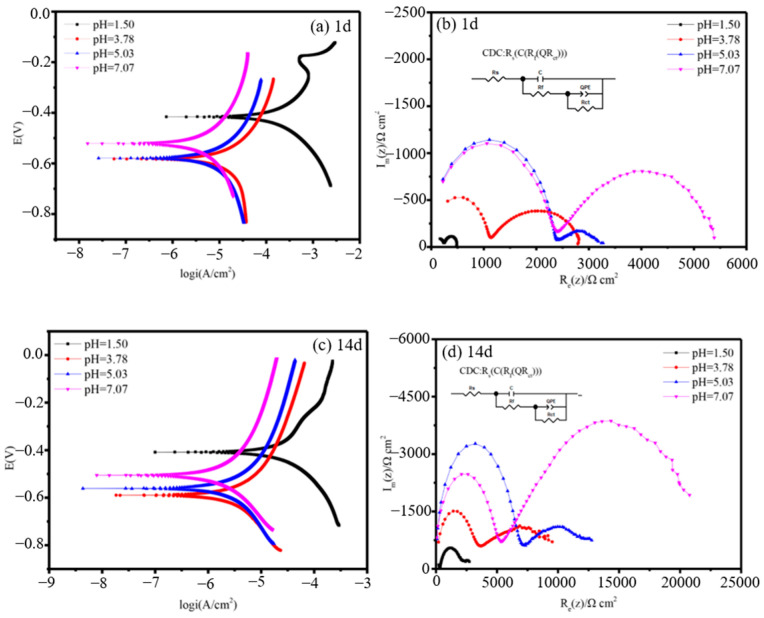
Polarization curves and EIS of X70 steel in the contaminated-silt system at pH values of 1.50, 3.78, 5.03, and 7.07: (**a**,**b**) 1 d; (**c**,**d**) 14 d [56].

The pH value, an important soil physicochemical characteristic, has an impact on the electrode potential of metals within the soil, ultimately influencing the corrosion rate of metals [49,50,51,52,53]. In the case of acidic and neutral soils, the corrosion rate of carbon steel increases as the pH value decreases [54,55]. The electrochemical corrosion behavior of X70 steel in contaminated silty soil with different pH values was tested by Han et al. [56]. As illustrated in Figure 8, the radius of the capacitive arc in the high-frequency region of X70 steel diminishes as the pH increases. Additionally, the corrosion rate and self-corrosion-current density decrease. Wu et al. [9] investigated the corrosion behavior of Q235 steel in simulated-soil solutions with varying pH values. The results revealed a direct relationship between the weight loss of Q235 steel in simulated-soil solution and the decreasing pH value. Furthermore, a rise in the cathodic-corrosion-current density and a shift towards more negative-corrosion-potential values were observed as the pH decreased. In highly acidic soils, the pH level plays a crucial role in the cathodic polarization process, specifically through the depolarization process of H^+^. Typically, in soils where the cathodic depolarization of oxygen predominates, the acidity of the soil affects cathodic polarization by counteracting the formation of OH^−^ generated during the cathodic process. In addition, the dissolution of metal ions through the anodic process leads to the formation of corrosion products with varying solubilities, influenced by the soil’s pH level. Hence, determining the pH value holds significance in comprehending the corrosiveness of the soil, as it influences numerous factors associated with corrosion in the soil environment.

### 4.3. Soil Resistivity

Soil resistivity holds significant importance in grounding engineering calculations as it directly influences various factors such as grounding resistance, ground potential distribution, contact voltage, and step voltage. It is commonly utilized as an indicator to assess the corrosiveness of soil [57]. Multiple factors contribute to soil resistivity, including mineral composition, moisture content, soil structure, and temperature. The concentration of conductive ions in the soil and the overall moisture content play a vital role in determining the soil resistivity [14,15,48]. A higher concentration of conductive ions in the soil results in better conductivity, while increased moisture content improves the electrical conductivity. However, it is worth noting that discussing the relationship between soil resistivity and corrosion becomes less meaningful when the soil’s water content is exceptionally low [6,44,49,54]. In terms of grounding systems, lower resistivity in the soil leads to more effective discharge of the grounding grid. Conversely, from the perspective of corrosion science, lower resistivity implies easier charge transfer, making the occurrence of corrosion more likely. Soil resistivity levels have been utilized as criteria for evaluating soil corrosiveness, as illustrated in Table 2.

### 4.4. Soluble Salt

The soluble-salt content in the soil has a direct impact on various physical and chemical properties of the soil. As the soluble-salt content increases, along with a rise in the concentration of charged ions, the soil becomes more electrically conductive and exhibits a lower resistivity. This conductivity enhancement is due to the presence of ions that can facilitate the flow of an electrical current in the soil. Moreover, changes in the concentration of the medium also influence the corrosion-current density. Therefore, the content of soluble salts in the soil not only affects its electrical properties but also has implications for the corrosion behavior of materials in contact with the soil.

**Figure 9:**
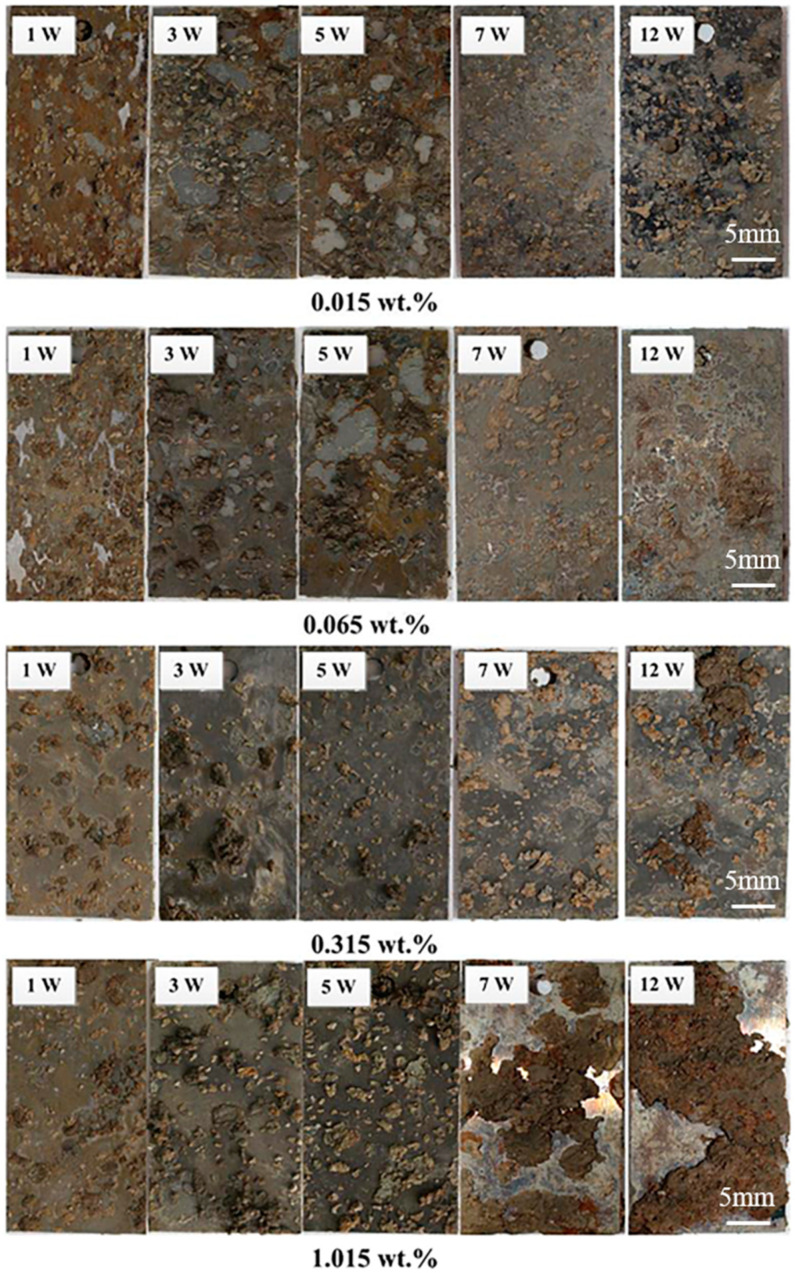
Carbon steel exposed to soil with different chloride ion concentrations for 1, 3, 5, 7, and 12 weeks [60].

The presence of soluble salts in soil has varying effects on soil corrosion. Among the corrosive ions in soil, ${Cl}^{-}$ and ${SO}_{4}^{2-}$ have the most influence on metal corrosion. ${Cl}^{-}$ is known for its small ionic radius, which accelerates the anodic corrosion process of metals and reacts with the metal matrix through the corrosion layer [44,59]. Studies conducted by Zhu et al. [14] have shown that an increase in ${Cl}^{-}$ content leads to higher corrosion-current density and weight-loss rate. Song et al. [60] investigated the corrosion performance of carbon steel in solutions with different chloride ion concentrations and found that the corrosion pattern varied with the chloride ion content. Small and substantial pitting corrosion can be observed at the initial point even when exposed to low chloride concentration. This phenomenon indicates that carbon steel is more susceptible to chloride attack and that chloride ions accelerate the corrosion of carbon steel (Figure 9). ${SO}_{4}^{2-}$, on the other hand, can affect the pH of soil, leading to increased acidity and reduced pH levels, indirectly contributing to soil erosion [61]. The ${SO}_{4}^{2-}$ content in soil also influences the mass transfer rate of the cathode and anode of electrode materials [55]. The corrosion effects of ${CO}_{3}^{2-}$ and ${HCO}^{-}$ on carbon steel differ. ${CO}_{3}^{2-}$ acts as a barrier to corrosion, while ${HCO}^{-}$ does not. Li et al. [62] investigated the corrosion behavior of Q235 steel in a simulated-soil fluid and found that an increase in ${HCO}^{-}$ and ${CO}_{3}^{2-}$ concentration led to the passivation of Q235 steel. Xie et al. [63] studied the corrosion behavior of X70 steel and observed that the corrosion-current density increased with higher ${HCO}^{-}$ concentration. ${CO}_{3}^{2-}$ plays a significant role in the corrosion of carbon steel, as it can react with ${Ca}^{2+}$ to form ${CaCO}_{3}$, which, in conjunction with sand particles in the soil, creates a strong protective layer. This layer effectively inhibits the anodic corrosion process and slows down the corrosion rate of carbon steel [64]. Soil cations exert minimal influence on corrosion due to their primarily conductive nature [65]. For instance, ions such as ${Ca}^{2+}$ tend to form insoluble carbonates when they are present in soil. These carbonates adhere to the metal surface, thereby retarding the corrosion process. Overall, the relationship between the total salt content of the soil and its susceptibility to erosion is shown in Table 3.

### 4.5. Temperature

**Figure 10:**
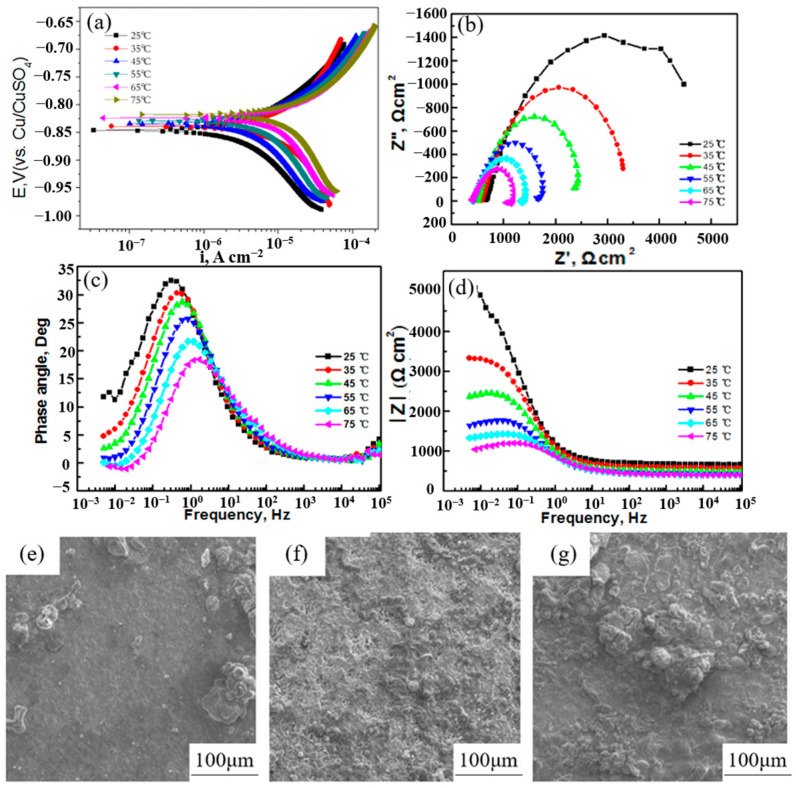
Potentiodynamic polarization curves (**a**) and EIS (**b**–**d**) of X80 steel after burying for 24 h in the temperature range 25 °C to 75 °C. SEM images of X80 steel after burying for 240 h at different temperatures: (**e**) 25 °C, (**f**) 55 °C, (**g**) 75 °C [69].

With variations in seasons, the effects of lightning inrush currents and the consequences of short-circuit incidents involving high currents, the temperature of the soil adjacent to the grounding grid undergoes substantial changes. This fluctuation in soil temperature triggers corresponding alterations in soil moisture content, resistivity, redox potential, oxygen content, and oxygen transmission capacity [48,67]. The elevation in temperature in the soil system accelerates both the diffusion process of the cathode and the ionization process of the electrochemical reaction. Furthermore, temperature directly impacts microbial activity, consequently leading to noteworthy transformations in soil erosion dynamics [7]. Through research conducted by Benmoussa et al. [54], the corrosion behavior of pipeline steel in a simulated-soil fluid was examined using techniques like kinetic potential polarization. The findings revealed an intensified corrosion tendency of steel, accompanied by an increase in corrosion-current density with rising temperature. Notably, soil resistivity declines as temperature rises. In general, an increase in temperature significantly accelerates the process of metal corrosion [68]. Wu et al. [69] investigated the effect of temperature on the corrosion of X80 steel in acidic soil, as illustrated in Figure 10. Within the temperature range of 25 to 75 °C, the charge transfer resistance decreased, while the corrosion-current density increased with the elevation of soil temperature. Furthermore, as the temperature rose, the corrosion products became loose and inhomogeneous, further aggravating the corrosion of X80 steel. Nie et al. [70] conducted an investigation into the electrochemical corrosion behavior of carbon steel in soil across varying temperatures. The results indicate that the anodic-current density of steel rises while the linear polarization resistance reduces with increasing temperature. At low temperatures, the steel passivates. However, as the temperature increases, the passivation current density increases, leading to a decrease in the passivation current interval. Moreover, the corrosion rate of steel increases with rising temperature, causing a decrease in impedance and charge-transfer resistance. However, the influence of temperature on the corrosion rate is more intricate for primary corrosion cells involving depolarization processes with oxygen. This complexity arises due to the fact that elevated temperatures accelerate oxygen diffusion while significantly lowering its solubility [71].

Soil temperature indirectly impacts soil erosion through influencing various other factors. One such factor is soil resistivity, which tends to increase with rising temperatures. Additionally, soil temperature also plays a role in the metabolic activity of microorganisms. It has been observed that microbial metabolism generally increases within a certain temperature range. These two factors, soil resistivity and microbial metabolism, are crucial in determining the extent of soil erosion.

### 4.6. Soil Oxygen Content

**Figure 11:**
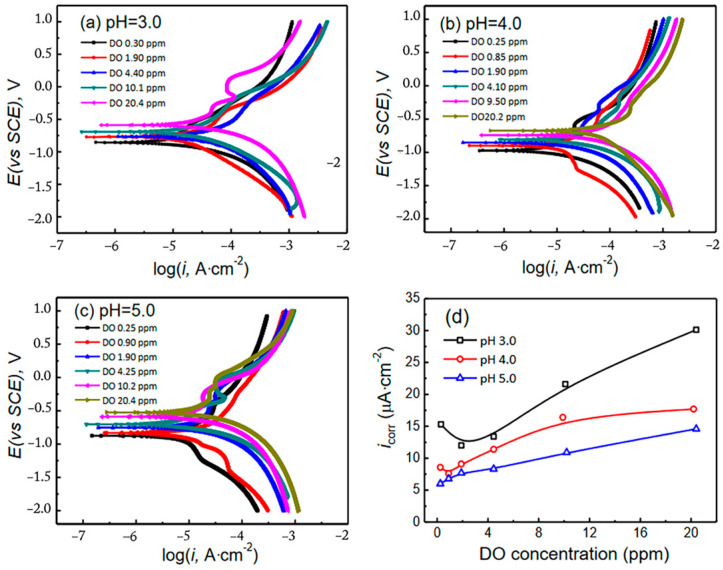
Polarization curves of X80 steel in soil-simulated solutions under different conditions: (**a**) pH 3.0; (**b**) pH 4.0; (**c**) pH 5.0. Corrosion-current-density fitting results (**d**) [72].

The oxygen levels in the soil are influenced by various factors, including soil type, moisture content, soil structure, particle size, inhomogeneity, etc. Oxygen concentration works in conjunction with other influencing factors to impact corrosion [16,71]. Some oxygen is present within the interstitial spaces among soil particles, while some dissolves in water in the soil. Generally, drier soil exhibits higher oxygen content, whereas moister soil has lower oxygen levels. The heterogeneous nature of soil leads to considerable variations in oxygen content within the same area, leading to the formation of oxygen concentration cells and subsequent corrosion. In cases where the backfill contains materials like gravel or construction debris, the limited permeability of the surrounding medium creates oxygen-deficient regions, acting as anodes within corrosion cells, while other metal components in the uniform soil act as cathodes. Xie et al. [63] conducted a study on the corrosion morphology of X70 steel at different dissolved-oxygen levels. The findings revealed that at higher dissolved-oxygen levels, corrosion manifested more severely, resulting in numerous corrosion pits on the surface. Conversely, as the dissolved oxygen decreased, both the number of corrosion pits and the corrosion rate diminished, indicating a deceleration in the corrosion process. Wang et al. [72] conducted a study on the corrosion behavior of X80 steel under varying dissolved oxygen (DO) concentrations and pH levels. Their findings indicate that the presence of oxygen accelerates the corrosion of X80 steel. At a pH of 5.0 (Figure 11c), the corrosion potential tends to increase with the increase in DO content, and the corrosion-current density increases with the increase in DO content.

**Figure 12:**
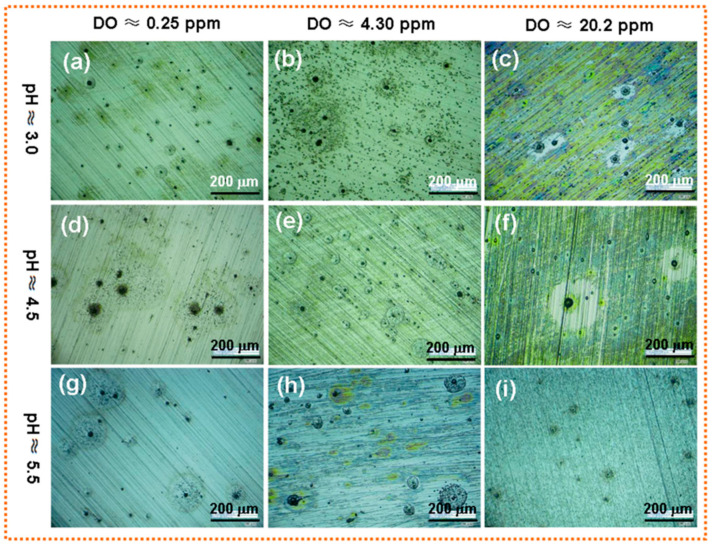
Surface OM image of X80 steel after EIS test in the acidic soil-simulated solution with various pH and DO contents. (**a**) pH ≈ 3.0 and DO ≈ 0.25 ppm; (**b**) pH ≈ 3.0 and DO ≈ 4.30 ppm; (**c**) pH ≈ 3.0 and DO ≈ 20.2 ppm; (**d**) pH ≈ 4.5 and DO ≈ 0.25 ppm; (**e**) pH ≈ 4.5 and DO ≈ 4.30 ppm; (**f**) pH ≈ 4.5 and DO ≈ 20.2 ppm; (**g**) pH ≈ 5.5 and DO ≈ 0.25 ppm; (**h**) pH ≈ 5.5 and DO ≈ 4.30 ppm; (**i**) pH ≈ 5.5 and DO ≈ 20.2 ppm [74].

In sandy soil, the size of soil particles plays a significant role in the soil’s oxygen content and can, therefore, impact the corrosion potential of carbon steel in the soil, as well as its cathodic reaction rate [39]. This, in turn, influences the corrosiveness of the soil. He et al. [73] investigated the effect of different sand-particle sizes on the corrosion behavior of X70 steel. The findings confirmed that as the grain size decreased the number and size of pitting pits increased, and the corrosion rate accelerated. Wang et al. [74] observed the corrosion morphology of X80 steel after EIS testing in soil-simulation solutions with different pH and DO contents, as shown in Figure 12. At the same pH, the corrosion of X80 steel increased with the increase of DO content. When the DO content was 4.30 ppm, the surface of the specimen showed a dense and high number of corrosion pits. In addition, the level of oxygen concentration in the soil affects the formation of corrosion products. A higher porosity facilitates the penetration of oxygen and water retention, while good permeability accelerates metal corrosion within the soil.

### 4.7. Microbial Corrosion

Microorganisms present in the soil may not be directly responsible for corrosion, but their biological activities have a direct or indirect influence on the corrosion process of metals [67,75]. Microbial metabolism produces inorganic acids, organic acids, sulfides, and hydrogen, which in turn enhance the cathodic polarization process of metal corrosion. These metabolites modify the oxygen concentration, salt content, and acidity of the metal’s surroundings, leading to the formation of localized corrosion cells, such as oxygen concentration cells [76,77,78]. Among these microorganisms, sulfate-reducing bacteria (SRB) and iron-oxidizing bacteria (IOB) have the most significant impact on corrosion performance. In practical operating conditions, it is primarily these two types of bacteria that accelerate material deterioration through synergistic action. IOB consumes oxygen in the medium, generating favorable conditions for anaerobic SRB growth and encouraging corrosion of the substrate with SRB.

SRB belong to a group of anaerobic microorganisms that can utilize sulfate or other oxidized sulfides as electron acceptors to dissimilate organic matter [79]. SRB can generate energy by reducing ${SO}_{4}^{2-}$ to $S^{2-}$. These bacteria are known to cause microbial corrosion of various industrial materials such as iron, mild steel, stainless steel, aluminum and aluminum alloys, copper and copper alloys, and nickel and nickel alloys [80,81]. In a study conducted by Sun et al. [82], the corrosion behavior of stainless steel in soil containing SRB was examined. The results showed a higher corrosion rate of steel reinforcement in soil with SRB compared to steel in soil without SRB. Corrosion can also be influenced by changes in oxygen availability. El Hajj et al. [16] demonstrated that the transition from aerobic to anaerobic conditions stimulated SRB activity and hydrogen sulfide ($H_{2}S$) production. This transformation led to the conversion of iron hydroxyl oxides formed during the aerobic phase to maghemite pyrite, resulting in improved steel protection under anaerobic conditions. Javaherdashti et al. [83] revealed that SRB can promote stress corrosion cracking in carbon steel. Li et al. [84] discovered that the biological activity of SRB alters steel corrosion by generating $H_{2}S$ and FeS films on the steel surface, exacerbating the corrosion process. Microorganisms have the ability to modify the electrochemical conditions on metal surfaces through the formation of biofilms, which can accelerate or inhibit corrosion [85].

**Figure 13:**
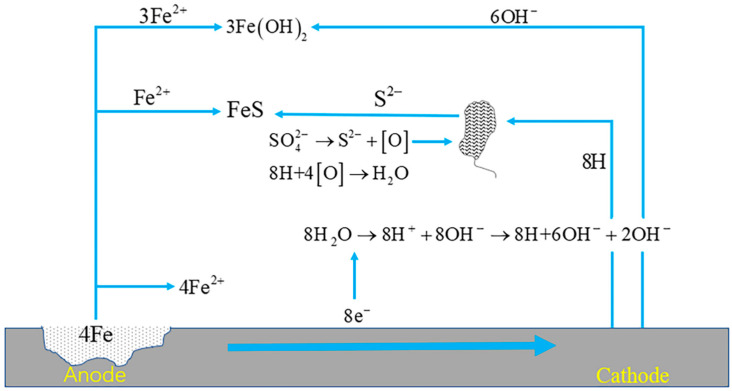
Corrosion mechanism of sulfate-reducing bacteria.

SRB can exert their corrosive effect through various mechanisms, including the cathodic depolarization mechanism, concentration cell mechanism, local cell mechanism, metabolite mechanism, acid-corrosion mechanism under sediment, and anodic-zone-fixation mechanism [86]. Among these mechanisms, the cathodic depolarization theory is widely recognized and accepted. According to the cathodic depolarization theory, SRB utilize ions present on the metal surface to reduce sulfate, leading to the production of sulfides. This process disrupts the normal electrochemical reactions occurring at the metal surface and contributes to the corrosion of the material. Figure 13 illustrates the corrosion mechanism associated with SRB. It is important to note that SRB can employ multiple mechanisms simultaneously, and the specific mechanism involved may vary depending on the environmental conditions and the type of material being corroded. Understanding these mechanisms is crucial for implementing effective corrosion-control strategies in environments where SRB activity is present.

### 4.8. Stray Current

**Figure 14:**
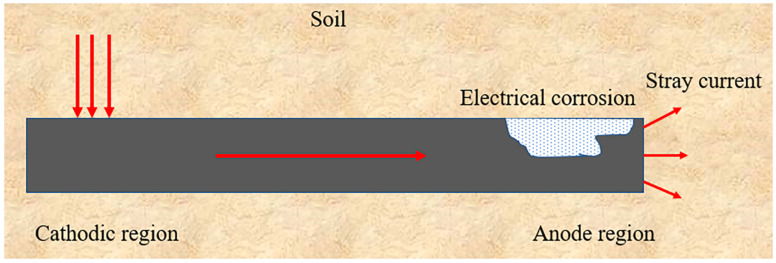
Stray-current corrosion mechanism.

The grounding grid, being exposed to underground environments for extended periods, is subjected to the influence of stray currents, which arise from the interference of alternating electric fields. It has been observed through numerous studies that stray currents can accelerate the corrosion rate and reduce the service life of grounding grids [14,87,88,89]. The corrosion mechanism induced by stray current on metal is depicted in Figure 14. The section where the stray current enters the grounding grid is protected as a cathode, while the area where it exits acts as an anode, accelerating corrosion. Stray currents accelerate the corrosion rate of the grounding grid and promote the deposition of corrosion products. Simultaneously, the effective dissipation area of the grounding grid diminishes due to the combined effects of ferrous metal corrosion dissolution and deposition of corrosion products. Consequently, the gradual accumulation of corrosion-product deposits obstructs the surface of the grounding grid, impeding the dissipation of ground current. When corrosion becomes severe or even leads to fractures, the dissipation of the grounding grid is further impeded, increasing the likelihood of ground faults.

**Figure 15:**
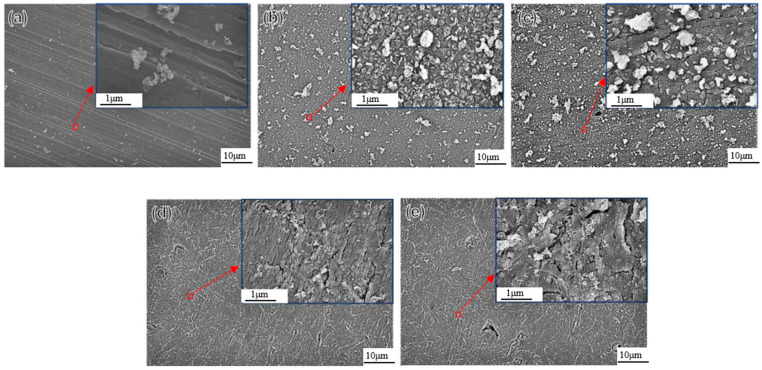
SEM corrosion-product images of X80 steel samples after 2 h in NS4 solution under various DC densities without strain: (**a**) i_DC_ = 0 mA/cm^2^, (**b**) i_DC_ = 0.25 mA/cm^2^, (**c**) i_DC_ = 0.5 mA/cm^2^, (**d**) i_DC_ = 1 mA/cm^2^, (**e**) i_DC_ = 2 mA/cm^2^ [94].

Currently, there are numerous studies that have investigated the impact of direct currents (DCs) on the corrosion behavior of carbon steel materials. The influence of DC stray currents in a soil environment is closely associated with the duration of the disturbance and the density of the stray current. It has been observed that the longer the stray current acts, the greater its effect on the corrosion rate [90,91]. Field exposure experiments and indoor tests at sites in Sweden and France by Sjogren et al. [92] demonstrated an increased corrosion tendency in stainless steel, carbon steel, and cast iron under the influence of stray DCs. Wang et al. [93] studied the corrosion behavior of X70 steel under the influence of direct currents. In the absence of an applied current, the specimen surface remained smooth without pitting pits. However, with an increase in DC density, the corrosion severity escalated, leading to the formation of numerous corrosion pits. Ma et al. [94] conducted a study to investigate the corrosion behavior of X80 steel in a soil-simulation solution under various stray DCs, as displayed in Figure 15. At a DC density of 0 mA/cm^2^, the corrosion rate was slow and exhibited a limited granular distribution (Figure 15a). As the DC density was increased to 0.25 mA/cm^2^, the corrosion-product layer became thicker than that observed without DC interference. When the DC density further increased to 0.5 mA/cm^2^ (Figure 15c), the corrosion products began to transform into those with a higher density. The corrosion rate escalated alongside the DC density, and the cracks on the corrosion-product layer multiplied significantly, exhibiting a loose and porous morphology (Figure 15e). Tan et al. [95] found that the presence of stray DCs significantly augmented the corrosion-current density and weight-loss rate of Q235 steel, Q235 galvanized steel, and Cu in soils with identical moisture-content. These findings highlight the substantial impact of stray DCs on the corrosion of carbon steel materials and emphasize the importance of managing and mitigating stray-current effects in practical applications.

**Figure 16:**
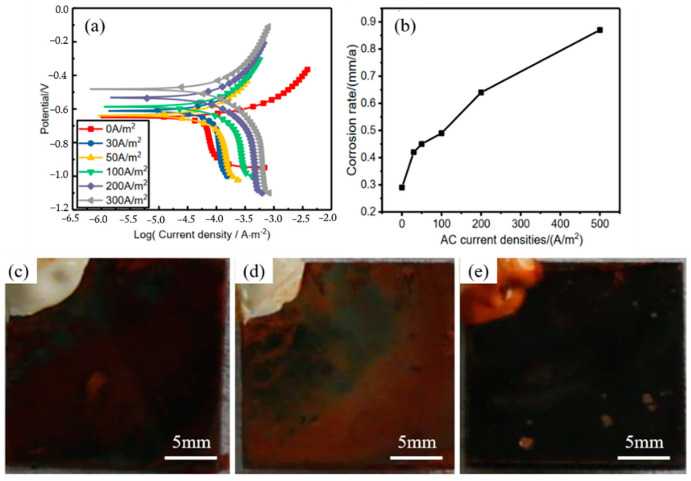
(**a**) Potentiodynamic polarization curves of X100 steel under various AC densities in simulated-acidic-soil solution. (**b**) Corrosion rates of X100 steel corroded in simulated-soil solution for 96 h at different AC densities. Optical images of the corrosion products of X100 steel in the simulated-acidic-soil solution after about 96 h under AC densities of (**c**) 0 A/m^2^, (**d**) 50 A/m^2^, and (**e**) 500 A/m^2^ [97].

When it comes to carbon steel grounding grids in soil, the grounding electrode is more susceptible to alternating current (AC) corrosion behavior, resulting in greater damage during the dissipation process due to its inherent current-dissipation characteristics. Fu and Cheng [96] investigated the AC corrosion behavior of pipeline steel in a solution using kinetic potential polarization tests and immersion tests. It was found that the presence of an alternating current led to a negative shift in the corrosion potential of pipeline steel and diminished its passivation ability in the solution. Similarly, Guo et al. [91] found a negative shift in the corrosion potential of X60 pipeline steel with increasing AC density, and the corrosion rate accelerated with higher anodic-current density. Furthermore, Yang et al. [97] conducted an experimental study on X100 steel under AC conditions, as depicted in Figure 16. Their findings indicate that both the corrosion rate and corrosion-current density increase with the rise in stray-current density. It is worth noting that even when cathodically protected, the presence of an AC can expedite material corrosion [98,99]. These findings emphasize the significance of addressing AC corrosion in carbon steel grounding nets and highlight the need for appropriate measures to mitigate its detrimental effects.

## 5. Corrosion Protection and Monitoring of Grounding Grid

### 5.1. Means of Protection against Corrosion of Grounding Grid

#### 5.1.1. Cathodic-Protection Method

**Figure 17:**
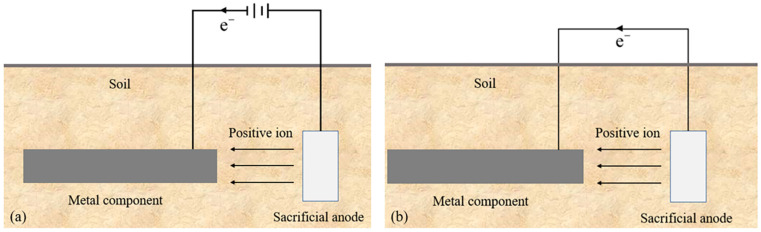
Cathodic-protection method: (**a**): applied-current method, (**b**): sacrificial-anode method.

Cathodic protection is a widely employed technique for safeguarding metallic structures against corrosion [100,101]. It consists of two main methods: sacrificial anode method and applied current method. Figure 17 illustrates the corrosion mechanism involved. In the sacrificial-anode method, a negative-charged auxiliary anode is connected to the ground grid to shield it from corrosion, with the more-negative metal corroding first, thereby safeguarding the grounding conductor. However, this method incurs the drawback of depleting the sacrificial-anode material and necessitating a loss of a greater amount. Conversely, the applied-current method employs an external power supply to provide an electrical current to the protected metal, inducing cathodic polarization and consequently retarding the corrosion rate [102]. The applied-current method serves as an economical and efficient means of protection [103]. However, its applicability is limited in situations with significant interference from stray currents and should be avoided in high-resistivity environments. Furthermore, there are certain drawbacks associated with this method, such as the lack of adjustability in the protection current, the inconvenience of online monitoring, and the intricate interconnection of the grounding grid with numerous steel structures on the ground. These factors impose several restrictions on the utilization of applied-current cathodic protection. In contrast, the sacrificial-anode approach finds extensive usage in protecting substation grounding grids. It not only provides cathodic protection but also reduces the resistance of the grounding system, facilitating the diffusion of industrial-frequency currents and lightning currents while eliminating the risks posed by stray currents. Although there have been some achievements in terms of the reliability and technical solutions of cathodic protection, it is important to note that the adoption of this method incurs high costs, and a comprehensive set of norms and standards is yet to be established.

#### 5.1.2. Resistance-Reducing Agent

The descending agent operates by leveraging its diffusion and penetration properties to diminish the resistivity of the soil surrounding the grounding system [104,105,106]. When a reducing agent is applied around the grounding body, it increases the effective cross-sectional area of the grounding system. Consequently, it attenuates soil corrosion on the grounding body to a certain extent, while simultaneously reducing the contact resistance between the grounding body and the soil, as well as the resistivity of the surrounding soil. This mechanism aids in enhancing the overall performance and effectiveness of the grounding system [107].

Currently, there are several types of drag-reducing agents used domestically, including chemical drag-reducing agents, physical drag-reducing agents, and rare-earth-class drag-reducing agents. Chemical drag-reducing agents primarily consist of electrolytes as their main conductive component, which results in stronger diffusion and penetration effects compared to other types of drag-reducing agents [108]. However, these agents are corrosive to metals, have poor stability and longevity, and can be easily washed away with rainwater. Physical drag-reducing agents, on the other hand, are non-electrolyte solid powder that utilize strong-alkali and weak-acid salts as the gelling materials. The descending material exhibits strong adsorption capabilities to the grounding body without containing electrolytes. Its conductivity remains unaffected by soil moisture content, and it offers better stability. Once solidified, it will not be lost due to changes in the water table [109]. Although it provides better anti-corrosive properties, the reduction in resistance is not as significant, and higher quantities of this agent are generally required. Rare-earth drag-reducing agents utilize the characteristics of rare-earth bentonite non-metallic minerals as their base material, with certain additives acting as drag-reducing substances [110]. This type of drag-reducing agent effectively lowers resistance, improves the resistivity of the surrounding soil, and has a minimal corrosive effect on the grounding grid, making it the most promising option. However, the construction process for using this agent is strict and more expensive compared to other types. Table 4 compares the performance of typical drag-reducing agents.

When selecting a descending resistive agent for a substation, it is important to consider the specific conditions and requirements of the substation. The selected agent should exhibit the following characteristics: low resistivity, low corrosion rate of the ground grid, long-lasting environmental protection, not contain water-soluble hazardous substances, and good stability. By considering these factors and selecting a resistive degradation agent that meets these criteria, the substation can ensure optimal performance, safety, and longevity of its grounding system.

#### 5.1.3. Conductive Anticorrosive Coatings

Conductive coatings typically consist of several key components, including base resin, conductive fillers, solvents, and additives. These coatings serve a dual purpose of providing conductivity and anticorrosive protection. When applied correctly, high-quality conductive anticorrosive coatings offer effective corrosion resistance against acids, alkalis, and salts [111,112,113].

**Figure 18:**
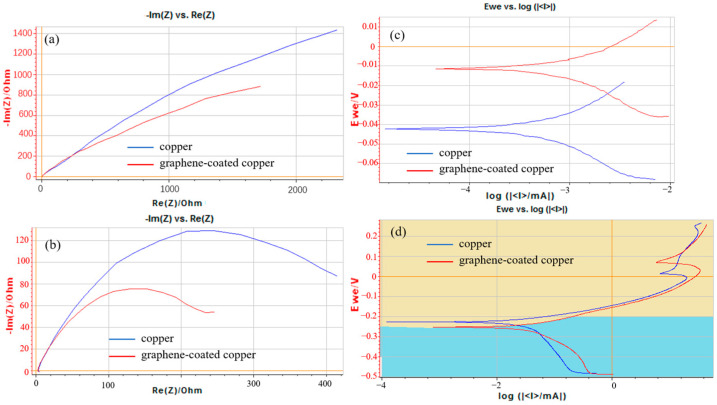
(**a**) Electrochemical impedance spectroscopy of bare and graphene-coated copper conductors in 0.5 M (0.5 molecular-weight gram powder in, per litre, volume of solution) Sodium sulphate solution. (**b**) Potentio electrochemical impedance spectroscopy of bare and graphene-coated copper conductors in 0.1 M sodium chloride solution. Polarization curves of the graphene-coated and uncoated copper in different solutions: (**c**) 0.5 M sodium sulphate solution; (**d**) 0.1 M sodium chloride solution [114].

**Figure 19:**
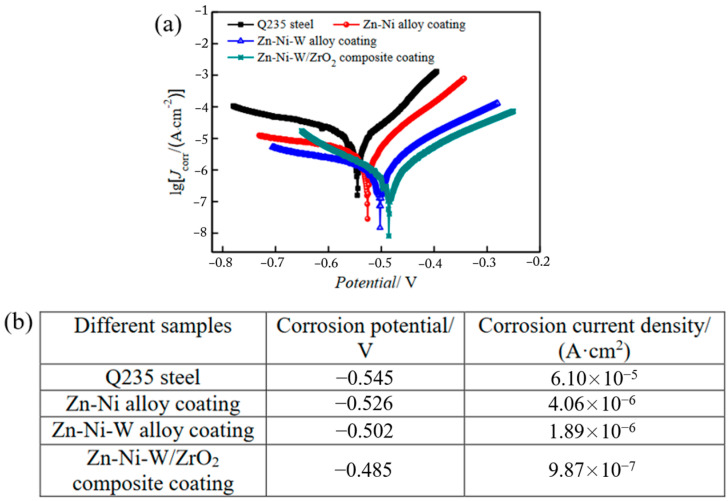
(**a**) Polarization curves of Q235 steel and different electrodeposited coatings in simulated-soil pore solution. (**b**) Fitting results of polarization curves [117].

In recent years, there has been significant progress in the research of conductive coatings. Datta et al. [114] conducted a study on the electrochemical corrosion behavior of both graphene-coated copper and bare copper in sodium chloride and sodium sulfate solutions, utilizing electrochemical impedance spectroscopy (EIS) and polarization curves (PC). The findings, as presented in Figure 18, demonstrate that graphene-coated copper displays superior corrosion resistance and reduced electrical resistance compared to bare copper. Wei et al. [115] investigated the long-term corrosion behavior of thermally sprayed stainless-steel-coated Q235 steel, Q235 steel, and galvanized steel in soil. It was found that Q235 steel experienced the most severe corrosion, with a substantial formation of corrosion products on its surface. Galvanized steel, though exhibiting better corrosion resistance than Q235, had its zinc layer deteriorated. On the other hand, the coated steel demonstrated the best corrosion resistance among the three materials, with the coating remaining intact on the specimen’s surface. Wu et al. [116] explored the corrosion resistance of Al-Si-coated Q235 steel and found it to be superior to that of galvanized steel under acidic and neutral conditions. However, its corrosion resistance decreased rapidly in acidic solutions. Polyaniline, characterized by its environmental stability, easy availability and high conductivity, has shown protective properties against various materials, such as carbon steel. Nonetheless, its low dispersion and adhesion in resin coatings significantly reduce the corrosion resistance of polyaniline composite coatings. Carbon nanotube-modified conductive coatings demonstrate promising applications. Zou et al. [117] conducted a study on the corrosion behavior of Q235 steel and various coatings in a simulated-soil solution using electrochemical tests, as illustrated in Figure 19. The results revealed that the application of different coatings effectively hindered the anodic and cathodic reactions of Q235 steel within the simulated-soil solution, thereby diminishing the corrosion propensity and corrosion rate of the steel. Li et al. [118] prepared multi-walled carbon nanotube/polyurethane composite coatings using electrostatic spraying and observed a reduction in corrosion-current density and a significant enhancement in corrosion resistance after applying the composite coating to the steel surface. During the construction process, coatings are susceptible to various defects such as pinholes, breaks, and scratches, which can accelerate corrosion. Ren et al. [119] suggested using conductive coatings in conjunction with cathodic protection as a corrosion-protection strategy. This combination not only slows down the aging rate of the coating, but also facilitates a more uniform distribution of a cathodic-protection current. As a result, the service life of the grounding device is significantly prolonged, providing enhanced protection against corrosion.

#### 5.1.4. New Grounding Materials

Conventional grounding materials encounter challenges such as poor corrosion resistance, high maintenance expenses, limited availability of resources, and environmental degradation. The innovation of novel grounding materials holds the potential to effectively mitigate these issues.

Stainless steel composites exhibit excellent electrical, mechanical, and thermal stability. They surpasses copper, galvanized steel, and other grounding materials in terms of corrosion resistance, while they are more cost-effective than copper and galvanized steel. The addition of appropriate alloying elements can enhance the corrosion resistance of the materials. Lv et al. [120] conducted a study on the corrosion behavior of high-silicon ferrochrome in soil, revealing that higher levels of silicon and chromium in the matrix significantly reduced the steel’s corrosion rate. Li et al. [121] developed a Cr microalloyed low-carbon steel and compared its corrosion behavior with that of Q235 steel in soil. The findings demonstrated that the corrosion resistance of Cr microalloyed low-carbon steel was greatly enhanced by incorporating a suitable amount of chromium, as compared to Q235 steel. Numerous metals encounter grounding issues, including corrosion and elevated grounding resistance. Graphite presents several advantages such as good electrical conductivity, low cost, and good stability. In recent years, it has emerged as a research focus for non-metallic conductive materials. Wang et al. [122] proposed a novel graphite grounding conductor with significantly lower grounding resistance and enhanced corrosion resistance when compared to traditional materials. This conductor is capable of withstanding sustained strong currents, among other benefits. Huang et al. [123] proposed a flexible graphite composite grounding material composed of high-purity flake graphite and conducted tests to confirm its adherence to electrical-engineering requirements. Furthermore, graphene-composite grounding devices have proven to possess superior current-drainage capacity and resistance-reducing effect compared to galvanized steel.

Graphite-composite grounding materials exhibit excellent grounding performance, particularly due to their superior corrosion resistance compared to metal grounding materials. This attribute significantly extends the service life of the grounding system and ensures operational safety for the grounding grid. As a result, graphite-composite grounding materials hold vast potential for diverse applications in the field of grounding-material development.

#### 5.1.5. Other Methods

Increasing the cross-sectional area of the grounding body has several advantages for the grounding grid. Firstly, it reduces power loss, resulting in improved overall grounding efficiency and performance, while also decreasing grounding resistance. Moreover, this increase in cross-sectional area has the potential to extend the lifespan of the grounding grid. However, it is important to consider that augmenting the cross-section necessitates greater material consumption and can pose challenges when it comes to welding.

### 5.2. Corrosion Diagnosis and Detection Methods for Grounding Grid

The grounding grid, being buried in the ground for extended periods, is subjected to harsh operating conditions, making corrosion problems unavoidable. Insufficient electrical connections between ground grid equalizing conductors or grounding leads can result in fault points, deteriorating the grounding grid’s performance and causing electrical-performance issues [124,125,126,127]. In severe cases, it can directly jeopardize the safe and stable operation of the power grid. Therefore, it is crucial to conduct comprehensive testing and analysis of the grounding grid’s condition. Various corrosion-assessment methods are commonly employed, including electrochemical analysis, electromagnetic-field analysis, and electrical-grid analysis. These techniques help evaluate the severity of corrosion and inform appropriate remedial measures [128].

**Figure 20:**
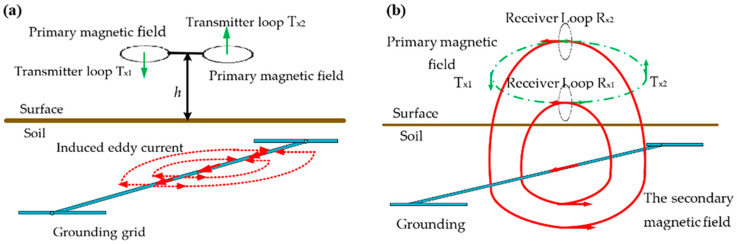
(**a**) The process of generating magnetic induction. (**b**) Detection process of secondary magnetic field [131].

*Electromagnetic-analysis methods.* Electromagnetic-field analysis is utilized to diagnose fault conditions in grounding grids. This approach involves examining the magnetic-field distribution on the ground surface, which can reveal abnormalities when the grounding conductor is broken or damaged [129]. By injecting a sinusoidal current of a different frequency into the grounding grid and detecting the resulting electromagnetic induction at the surface, a comparison with the pre-energization magnetic-field-strength distribution can determine the corrosion state of the grounding grid. Operating parameters of the grounding grid during operation are measured using known grid topology and the fundamental concept of corrosion detection [130]. Wang et al. [131] proposed a specific detection method based on magnetic-field excitation, as shown in Figure 20. By accurately positioning the coil, the secondary magnetic-field signal is extracted from two magnetic fields, enabling precise measurement of underground metal corrosion. This non-contact method provides higher measurement accuracy and stronger anti-interference capabilities compared to traditional methods and is not restricted by the grounding grid’s structure. In cases where the grounding grid drawings are missing or the actual structure of the grounding grid differs from the provided drawings, magnetic-field inspection can be a helpful solution for detecting grounding grid corrosion. The method is simple and effective; however, when corrosion is not severe and there are no fractures present, the magnetic field changes may not be apparent, resulting in less-accurate detection results.

**Figure 21:**
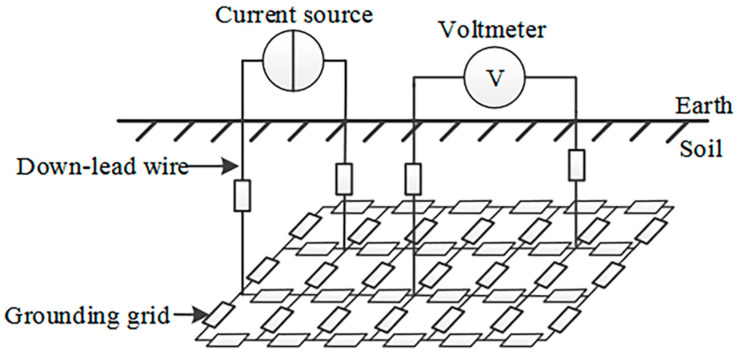
Equivalent physical model of the grounding network [132].

*Electrical-grid-analysis methods.* Electrical-grid-analysis methods involve simplifying the grounding grid into a purely resistive grid [132]. The equivalent physical model of this approach is shown in Figure 21. By applying DC excitation to the grounding grid and measuring the corresponding response parameters of the port, the fault-diagnostic equations can be established to determine the actual resistance value. The degree of corrosion in each conductor section can then be diagnosed by comparing it with information such as the initial resistance value obtained during the construction program [133]. The electrical-grid-analysis method has a long history of research, and the theory behind it is relatively well developed. However, this method has limitations regarding the location and number of underground wires, making it difficult to apply to medium and large grounding grids.

*Electrochemical Method.* The corrosion process of grounding grid conductors primarily involves electrochemical reactions influenced by factors such as air, soil pH value, and moisture content. Huang et al. [134] developed an electrochemical method for detecting the depth of corrosion in grounding bodies, demonstrating its accuracy in measuring this parameter, and indicating the overall state of the grounding body. With the advancement of electrochemical theory, various methods such as the linear polarization method and constant-current step have emerged, allowing for the evaluation of the grounding grid’s condition by direct-measuring electrochemical parameters and converting them into corrosion rates [135]. The electrochemical method is relatively straightforward to operate, provides fast detection, and can directly reflect the corrosion rate of the grounding grid. However, further research is required to address signal interference at the testing site and to enhance the design performance of the sensors used in this method.

## 6. Conclusions and Prospects

In conclusion, the corrosion of grounding grids is influenced by various soil properties and factors such as resistivity, moisture content, pH value, oxygen content, and soil temperature. These factors interact with each other, and their combined effect determines the extent of corrosion. Microorganisms and stray currents in the soil can accelerate corrosion directly or indirectly. Although galvanized and cathodic protection methods are commonly used for protection at present, they have limitations and provide only limited protection against corrosion. To address these challenges, future research and development can focus on the following prospects:

1. Exploration of new materials: research and development of new materials with excellent corrosion resistance, such as graphene-composite grounding materials, carbon-fiber-based-composite grounding materials, and graphite/glass-fiber-composite grounding materials, can replace traditional materials and extend the service life of grounding grids.
2. Environmentally friendly anti-corrosion technologies: Investigation of environmentally friendly anti-corrosion treatments (e.g., research on degradable anti-corrosion agents, low-energy, and low-pollution anti-corrosion treatments), can reduce environmental pollution and hazards. Meanwhile, it is crucial to further investigate and innovate in the field of grounding-grid-material recycling. Further advancements in coating anti-corrosion technology, including improving adhesion, wear resistance, and overall corrosion-resistance performance, can be beneficial. Additionally, combining anticorrosive coatings with cathodic protection and other protective measures can be explored.
3. Integration of intelligent technology: Through combining intelligent technology, methods and equipment for real-time monitoring of grounding grid corrosion can be developed. This would enable timely monitoring and early warning of corrosion, thereby enhancing the operational stability and reliability of the grounding grids.

Through pursuing these prospects, we can address the challenges posed by grounding grid corrosion and work towards improving their longevity and performance.

## Figures and Tables

**Table 1:** Comprehensive comparison of typical grounding grid materials.

Material	Price (Ten Thousand CNY/Ton)	Corrosion Resistance	Maintenance Fees/Remodeling Possibilities	Life Expectancy	Shortage
Carbon steel	0.35	worst	high maintenance/requires extensive excavation	<10	poor corrosion resistance
Galvanized steel	0.5	worse	high maintenance/requires extensive excavation	10–15	poor corrosion resistance
Copper-plated steel	2.5	better	no maintenance	30–50	poor corrosion resistance in acidic soils
Copper (>99.9%)	6.0	best	no maintenance/no remodeling required	50	expensive and polluting

**Table 2:** Relationship between soil resistivity and corrosivity [58].

Soil Resistivity, Ω·m	Soil Corrosivity	The Average Corrosion Rate of Steel, mm·a^−1^
0~5	very high	>1
5~20	high	0.2~1
20~100	moderate	0.05~0.2
>100	low	<0.05

**Table 3:** Correspondence between soil salt content and corrosivity [66].

Soil Salt Content (%)	Soil Corrosivity
<0.05	very low
0.05~0.2	low
0.2~0.5	moderate
0.5~1.2	high
>1.2	very high

**Table 4:** Comparison of typical resistance-reducing agents.

Types of Drag-Reducing Agent	Advantage	Shortage
chemical drag-reducing agent	strong penetration and diffusion	poor stability and service life [108]
physical drag-reducing agent	stronger adsorption to grounding grid	reduction of resistance is not significant [109]
rare earth class drag-reducing agent	less corrosive and more effective on the grounding grid	highly demanding and costly construction [110]

## Data Availability

Not applicable.
